# Investigation of optimal convolutional neural network conditions for thyroid ultrasound image analysis

**DOI:** 10.1038/s41598-023-28001-8

**Published:** 2023-01-24

**Authors:** Joon-Hyop Lee, Young-Gon Kim, Youngbin Ahn, Seyeon Park, Hyoun-Joong Kong, June Young Choi, Kwangsoon Kim, Inn-Chul Nam, Myung-Chul Lee, Hiroo Masuoka, Akira Miyauchi, Sungwan Kim, Young A. Kim, Eun Kyung Choe, Young Jun Chai

**Affiliations:** 1 Department of Surgery, Gachon University College of Medicine, Gil Medical Center, Inchon, Korea; 2 Transdisciplinary Department of Medicine and Advanced Technology, Seoul National University Hospital, Seoul, Korea; 3 Department of Surgery, Seoul National University Bundang Hospital, Seongnam-si, Gyeonggi-do, Korea; 4 Department of Surgery, College of Medicine, The Catholic University of Korea, Seoul, Korea; 5 Department of Otolaryngology-Head and Neck Surgery, College of Medicine, The Catholic University of Korea, Seoul, Korea; 6 Department of Otorhinolaryngology-Head and Neck Surgery, Korea Cancer Center Hospital, Korea Institute of Radiological and Medical Science, Seoul, Korea; 7 Department of Surgery, Kuma Hospital, Kobe, Japan; 8 Department of Pathology, Seoul Metropolitan Government Seoul National University Boramae Medical Center, Seoul, Korea; 9 Department of Surgery, Seoul National University Hospital Healthcare System Gangnam Center, Seoul, Korea; 10 Department of Surgery, Seoul Metropolitan Government, Seoul National University Boramae Medical Center, 20 Boramaep-ro 5-gil, Dongjak-gu, Seoul, 07061 Korea

**Keywords:** Image processing, Machine learning, Endocrinology

## Abstract

Neural network models have been used to analyze thyroid ultrasound (US) images and stratify malignancy risk of the thyroid nodules. We investigated the optimal neural network condition for thyroid US image analysis. We compared scratch and transfer learning models, performed stress tests in 10% increments, and compared the performance of three threshold values. All validation results indicated superiority of the transfer learning model over the scratch model. Stress test indicated that training the algorithm using 3902 images (70%) resulted in a performance which was similar to the full dataset (5575). Threshold 0.3 yielded high sensitivity (1% false negative) and low specificity (72% false positive), while 0.7 gave low sensitivity (22% false negative) and high specificity (23% false positive). Here we showed that transfer learning was more effective than scratch learning in terms of area under curve, sensitivity, specificity and negative/positive predictive value, that about 3900 images were minimally required to demonstrate an acceptable performance, and that algorithm performance can be customized according to the population characteristics by adjusting threshold value.

## Introduction

Ultrasound (US) is the first-line imaging modality used in the screening and diagnosis of thyroid nodules and cancer. However, the wide range of reported inter-operator accuracy (29–84%)^1,2^ remains a concern, despite improved image resolution and intensive efforts to standardize diagnoses through initiatives such as the Thyroid Imaging Report and Data System^3^. To overcome the high operator-dependent nature of US imaging of thyroid nodules, beginning in 2008 artificial intelligence (AI) trained on large image datasets has been applied to recognize complex patterns and produce quantitative assessment^4^. The use of AI models trained on neural networks is appropriate because the characteristics of a thyroid nodule can be captured in one representative US image.

Large volumes of labeled images are required for training AI models because neural network performance improves with the training dataset size^5^. However, labeled medical data is difficult to acquire due to privacy constraints on work with health records as well as the labor intense nature of data labeling^6^. Data augmentation and transfer learning can help overcome these limitations. Data augmentation artificially generates more images by altering the physical properties of the original images such as width to height ratio, noise, and color, or horizontally flipping them. Although this tactic is reported to improve the performance of algorithms^7^, when applied to thyroid images, augmentation of images may distort the essential characteristics of nodule shape, echogenicity, margin, and calcification of thyroid nodules, which are necessary for accurate US diagnosis of thyroid nodules^8^. Transfer learning may be a more suitable for deep learning using medical image^9^, especially in thyroid US images^10^ as well. Transfer learning is a common method in computer vision because it can achieve high accuracy in a short time^11^. Unlike scratch learning which requires a model to be taught from the beginning of the project based on random weights, transfer learning applies knowledge that has already been gained from one task (source task) to a different task (target task). ImageNet is an image database organized according to the nouns of the WordNet hierarchy, in which each hierarchy is associated with more than one hundred images. ImageNet dataset’s pre-trained models are one of the most popular base datasets. While scratch learning training models use only images acquired for a specific project, the transfer learning approach uses pre-trained neural networks and is generally more efficient despite using datasets that do not include the same type of images^9^. However, there are not enough large scale thyroid US imaging studies directly comparing scratch vs. transfer learning models that have been reported^12,13^. Such transfer learning methods have been successfully applied in other image modalities such as X-ray, and computed tomography^14,15^. Furthermore, the optimal number of images required to successfully train a neural network model using transfer learning techniques is unknown. As studies use larger datasets to improve the performance of algorithms, it would be useful for medical researchers to be able to predict the outcome of the full data before training the model with the full dataset, especially as medical data is difficult to acquire.

Finally, the setting of a diagnostic tool should be adjusted according to the characteristics of the population that it is screening. For example, the sensitivity and specificity of a diagnostic tool must be set according to whether malignancy is widespread or rare within the population screened. To the best of our knowledge, deep learning training research has not yet reported the adjustment of an algorithm’s performance to such population characteristics.

The aim of the current study was to compare the efficacy of our scratch learning-based and transfer learning-based deep learning algorithm models in distinguishing between benign and malignant thyroid nodule US images. Additionally, we conducted stress tests to determine the proportion of original data required to efficiently train the deep learning algorithms and customized the threshold level to reflect varying diagnostic characteristics of target populations.

## Methods

### Dataset collection

**Figure 1:**
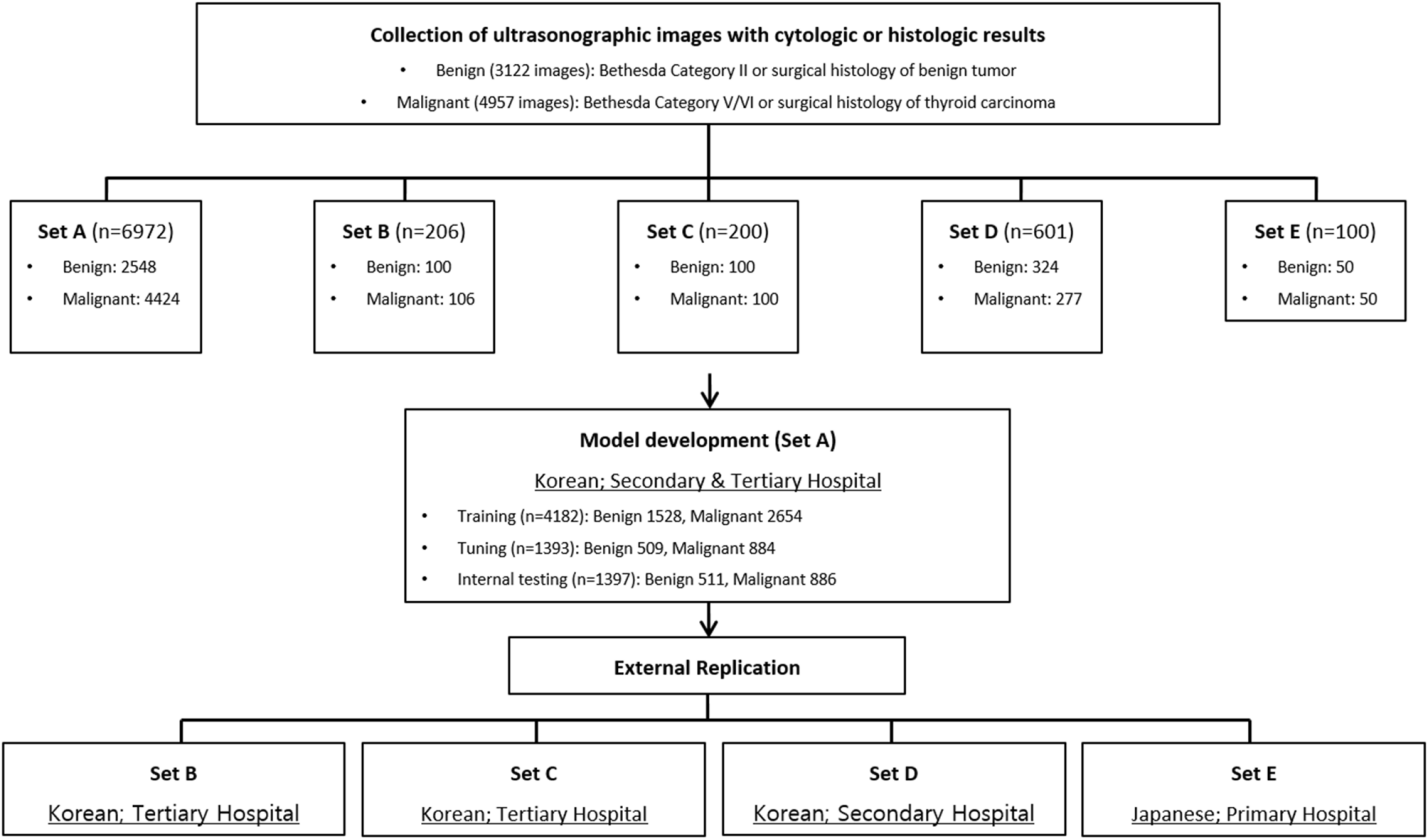
Flow chart of data collection and composition.

**Figure 2:**
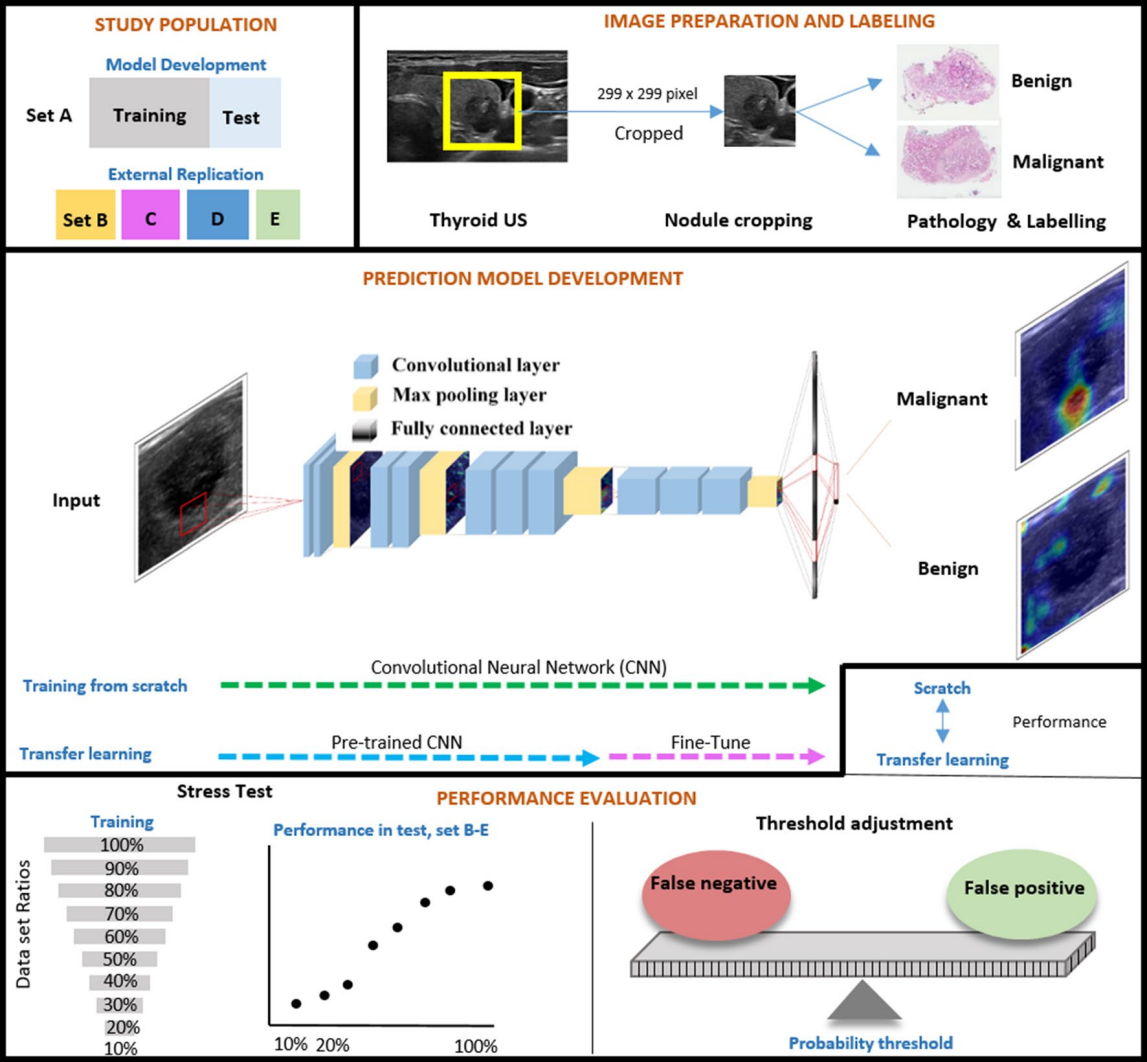
Overview of the study design.

Figure 1 shows the flow chart of data collection and its composition. US images were collected from the records of patients who underwent surgery or fine needle aspiration cytology examination for thyroid nodules. From these data, we developed a model to predict the pathology of thyroid nodules (malignant vs. benign) using features of US images. We used data from two medical institutions (Seoul Metropolitan Government Seoul National University Boramae Medical Center and Seoul National University Bundang Hospital) for model development (Set A in Fig. 1). The training set consisted of 4182 thyroid US images (1528 benign, 2654 malignant), the tuning set consisted of 1393 thyroid US images (509 benign, 884 malignant), and the internal test set consisted of 1397 images (511 benign, 886 malignant). Images were stored in Digital Imaging and Communications in Medicine (DICOM) file format. For external replication of the developed model, we used data from four institutions to overcome the issue of overfitting (Set B, Incheon St. Mary’s Hospital, Korea; Set C, Seoul St. Mary’s Hospital, Korea; Set D, Korea Cancer Center Hospital, Korea; and Set E, Kuma Hospital, Japan). The institutions were different sizes, and three sites primarily treat Korean patients while one primarily treats Japanese patients. With the collected images, the study was designed as according to Fig. 2.

### Image preparation

DICOM files were transferred to Portable Network Graphic files and the images of thyroid nodules were cropped into squares. For consistent cropping, we developed a web-based in-house program using JavaScript software. Clinicians cropped the region of interest into a square using mouse drag. The square size was set at a minimum of 299 × 299 pixels in order to obtain sufficient resolution. Cropped images larger than 299 × 299 pixels were downsized to 299 × 299 pixels. The process is further described in Supplementary Fig. 1.

### Image labeling

One experienced clinician (Y.J.C) labeled the images as benign (fine needle aspiration cytology Bethesda Category II or surgical histology of benign tumor) or malignant (fine needle aspiration cytology Bethesda Category V/VI or surgical histology of thyroid carcinoma).

### Model development

Supplementary Fig. 2 shows a general schematic map of the convolutional neural network architecture utilized in the proposed framework for prediction of benignity or malignancy of thyroid nodules. All models in this study (i.e., stress test, scratch-based, and ImageNet-based) were trained under the same conditions. VGG16^16^, VGG19^10^, and ResNet50^17^ were selected as classification architectures to validate transfer learning. The VGG16 network contained 13 convolutional, 13 activation, four pooling, and three full-connection layers. The VGG19 network contained 16 convolutional, 16 activation, four pooling, and three full-connection layers. The structure of ResNet allowed the gradients to flow backward directly through an identity connection from the later layers to the initial filters. After a set of convolution layers of each model, 1024 features with the same structure were extracted (average pooling, fully connected layer, and dropout; p = 0.5) and trained to predict malignancy or benignity through those features. Thereafter, 1024 features with the same structure were extracted (Average pooling, Fully connected layer, and Dropout; p = 0.5) and trained to predict malignancy or benignity through those features. All experiments were conducted using the NVIDIA RTX3090 GPU set-up with 300 epochs and a batch size of 16. For the hyperparameter control experiment, the initial learning rate was set to 0.00005, the optimizer was set to stochastic gradient descent (momentum = 0.9), and categorical cross entropy loss function was used. Data augmentation techniques such as zoom (− 0.1 to 0.1), rotation (− 5$^\circ$ to  + 5$^\circ$), and width/height shift (− 0.1 to 0.1) were used to create more images to train the model efficiently. Versions for program language and deep learning platform were Python 3.5 and PyTorch 1.12 with CUDA 10.2.

### Performance evaluation

#### Performance measurements

We compared the performance of each model with the test set and the four external replication sets. The performance of each model was evaluated using area under the receiver operating characteristic curve (AUC), accuracy, sensitivity, specificity, positive predictive value (PPV), and negative predictive value (NPV). Performance is shown as mean and standard deviation.

#### Performance comparison between scratch vs. transfer learning

We compared the performance of the transfer learning and scratch learning models using three neural networks (VGG16, VGG19, and ResNet50) on thyroid US image datasets.

#### Stress test

We conducted stress tests to determine whether the training dataset was large enough to saturate the error rate on the validation set. We designed an experiment using different dataset ratios at 10 ~ 100% (in 10% intervals) of the total training set. For assumption of real environment, we randomly selected benign and malignant samples in the same proportion as the total training set. For each interval, 10 tests were performed for each internal and external replication set using the three neural networks.

#### Threshold adjustment

We further tested whether the performance of the algorithm varied according to adjustment of the probability threshold. We tested the performance of the algorithms at the following threshold settings: 0.3, 0.5, and 0.7.

### Statistical evaluation

For statistical analysis of stress tests and experiments on comparison of initial weights, we compared the average AUCs and performed a paired t-test of US image datasets with classification models in internal and external validation sets. Data was analyzed using SPSS Statistics for Windows, version 28 (IBM Corp, Armonk, NY). For the stress test, paired t-tests were used for the intragroup comparison of AUC values of the 100% ratio-trained model and those of each of the models trained with 10–90% ratios of the training datasets. We also performed a comparative analysis of AUC, accuracy, sensitivity, specificity, PPV, and NPV by classifier threshold using each model trained on 100% of the training set for statistical analysis of models with initial weights learned in different domains.

### Ethical approval

The institutional review boards of all participating institutions approved this study. Representative institutional review board approval was granted by Seoul Metropolitan Government Seoul National University Boramae Medical Center (H-10-2020-195), and the study was conducted in accordance with the Declaration of Helsinki. Informed consent was waived by the board. The manuscript was written in concordance to the Strengthening the Reporting of Observational Studies in Epidemiology guidelines^11^.

## Results

### Scratch vs. transfer learning model (probability threshold 0.5)

The scratch and transfer learning models were compared individually with the performances of the internal test set (Set A) and four external datasets (Sets B–E). External replication Set B (Incheon St Mary hospital) contained 100 benign and 106 malignant thyroid US images, Set C (Seoul St Mary hospital) contained 100 benign and 100 malignant thyroid US images, Set D (Korea Cancer Center hospital) contained 324 benign and 277 malignant thyroid US images, and Set E (Kuma hospital) contained 50 benign and 50 malignant thyroid US images (Fig. 1). The probability threshold value was set to 0.5 for the test and replications. Although there were no significant differences in performance between the three architectures, VGG19 tended to demonstrate more statistically significant results than the others. Therefore, we present our results based on the VGG19 backbone. The VGG and ResNet results are separately summarized in the supplementary materials (Supplementary Tables 1–5).

**Figure 3:**
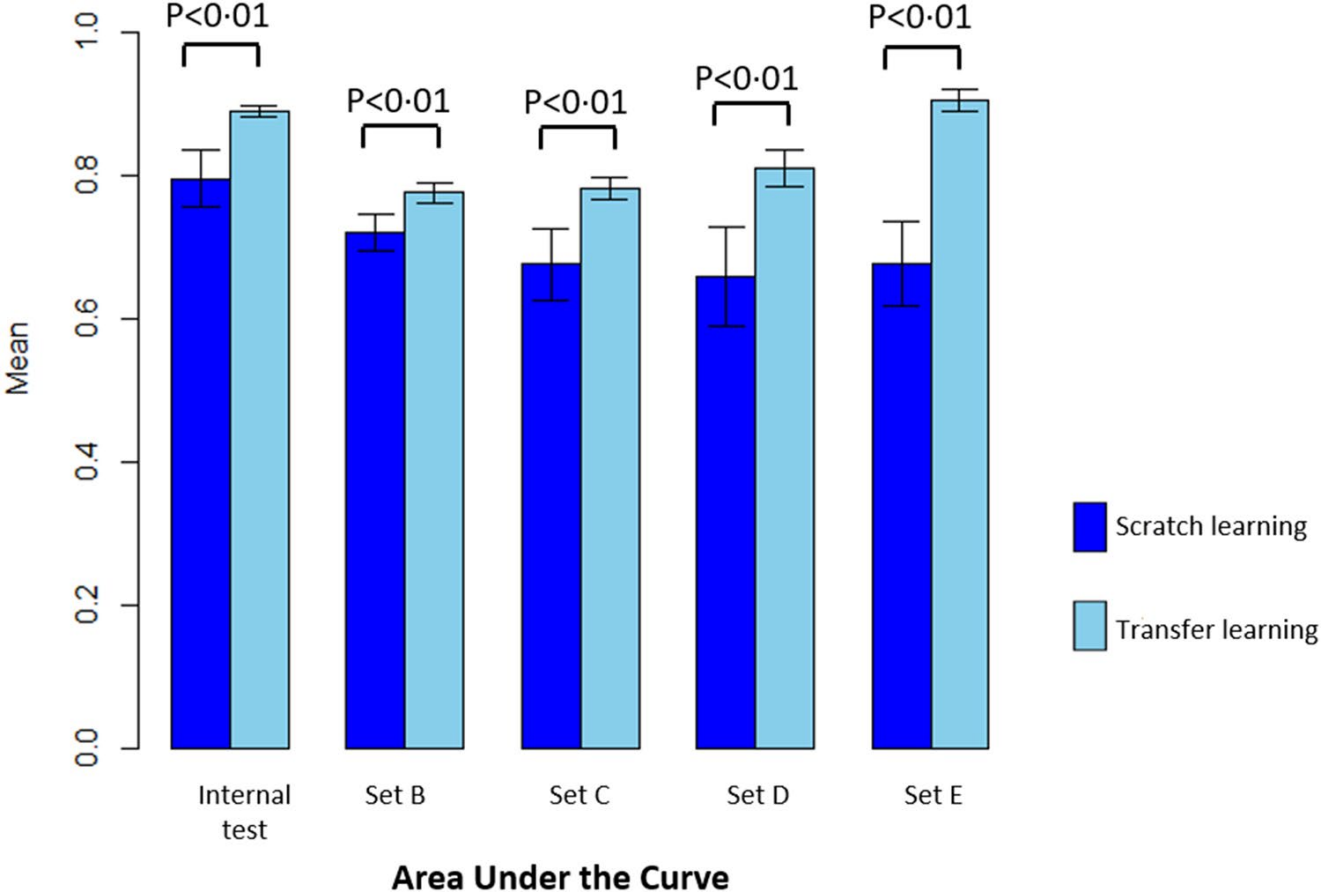
Comparing the mean AUC with standard deviation between scratch learning and transfer learning in five data sets.

The AUC values of the scratch vs. transfer learning models with the probability threshold set at 0.5, are represented in Table 1 and Fig. 3. All validation results indicate that the transfer learning model had a significantly higher performance than the scratch model.

**Table 1:** Comparison of the performance between scratch and transferred learning models.

Type of learning	Datasets	AUC	Accuracy	Sensitivity	Specificity	PPV	NPV
Scratch learning	Internal test set A	0.795 ± 0.039	0.735 ± 0.034	0.808 ± 0.028	0.607 ± 0.061	0.782 ± 0.029	0.646 ± 0.049
	External replication set B	0.720 ± 0.026	0.658 ± 0.020	0.807 ± 0.038	0.500 ± 0.072	0.633 ± 0.025	0.711 ± 0.020
	External replication set C	0.675 ± 0.049	0.632 ± 0.035	0.736 ± 0.056	0.527 ± 0.054	0.609 ± 0.029	0.668 ± 0.049
	External replication set D	0.658 ± 0.070	0.566 ± 0.035	0.878 ± 0.067	0.299 ± 0.091	0.518 ± 0.023	0.753 ± 0.08
	External replication set E	0.676 ± 0.058	0.528 ± 0.024	0.986 ± 0.02	0.07 ± 0.048	0.515 ± 0.013	0.66 ± 0.395
Transferred learning	Internal test set A	0.889 ± 0.007	0.816 ± 0.012	0.854 ± 0.037	0.751 ± 0.063	0.858 ± 0.027	0.752 ± 0.035
	External replication set B	0.775 ± 0.015	0.687 ± 0.017	0.803 ± 0.054	0.564 ± 0.070	0.664 ± 0.023	0.734 ± 0.035
	External replication set C	0.781 ± 0.015	0.692 ± 0.02	0.868 ± 0.05	0.517 ± 0.076	0.644 ± 0.023	0.805 ± 0.048
	External replication set D	0.809 ± 0.026	0.582 ± 0.050	0.979 ± 0.019	0.242 ± 0.108	0.527 ± 0.033	0.941 ± 0.032
	External replication set E	0.905 ± 0.016	0.634 ± 0.064	0.984 ± 0.02	0.284 ± 0.145	0.584 ± 0.055	0.96 ± 0.041

The sensitivity results of the scratch vs. transfer learning models are described in Table 1. Apart from the Set B dataset, the transfer learning model demonstrated superior outcomes. The NPVs for the scratch vs. transfer learning model indicated superior performance of the transfer learning model. This trend was also evident for accuracy and PPVs, without statistical significance. However, specificity was inconsistent between sets: the transfer learning model performed best for the test set and Set B datasets, while the scratch model performed best for Sets C-E datasets (Fig. 2, Supplementary Fig. 3).

### Stress test

**Figure 4:**
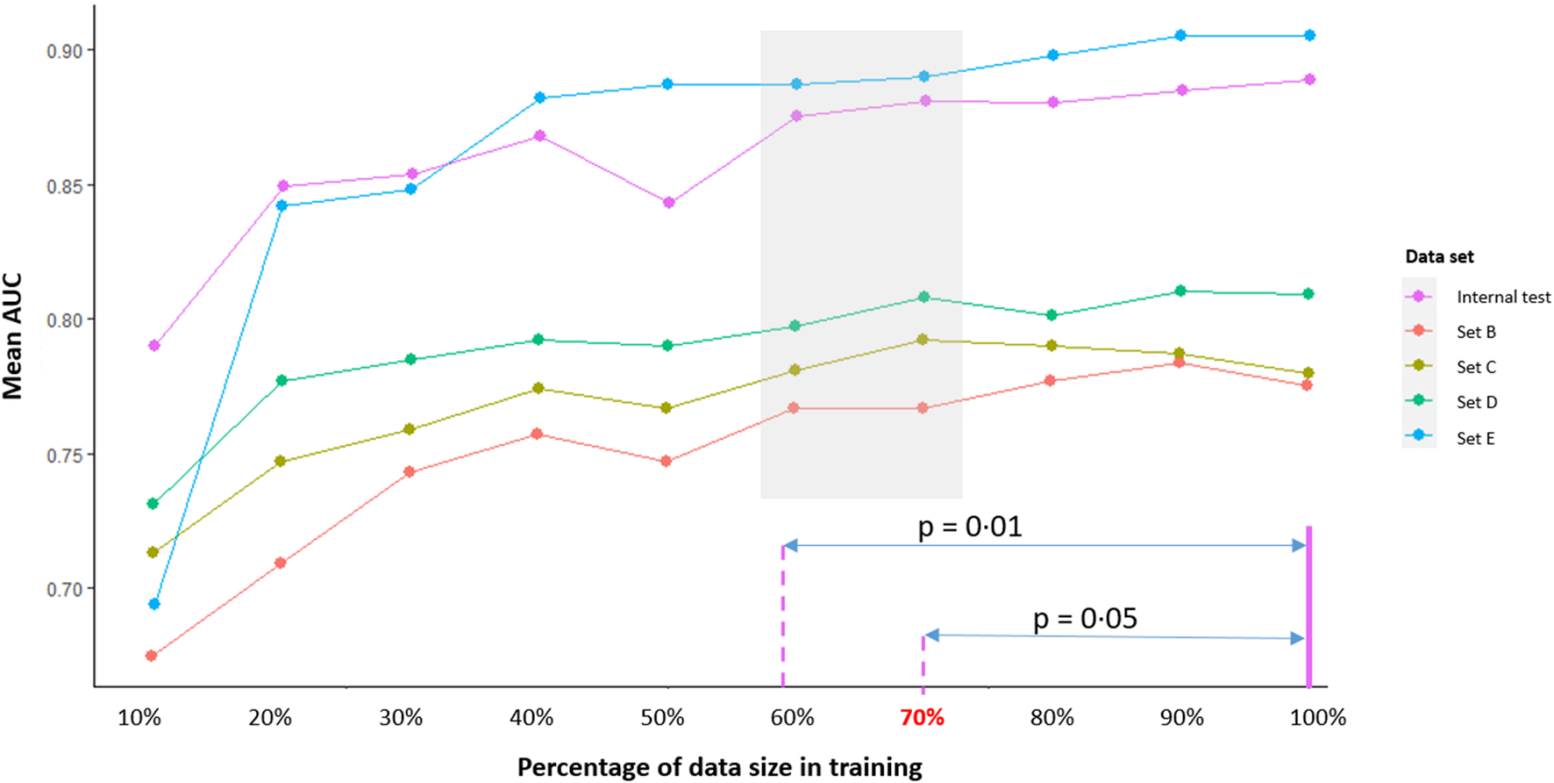
Stress test; changes of performance according to the increment of data size in training set.

The results of the stress test which was conducted in 10% increments are summarized in Fig. 4. For each architecture, as the number of cases increased, the AUC tended to increase in the internal and all four external datasets. In general, we were able to discern a trend showing that performance saturated at around 70% (3902 images) of the total dataset (5575). When validated against the 100% dataset, performance at 70% or above the total data was not statistically different. However, the performance of the datasets using 60% or below of the total data was significantly lower than that of the 100% dataset (Fig. 4). This indicates that 70% of the original dataset was the minim required to efficiently reproduce a comparable outcome to the 100% dataset results.

### Probability threshold 0.3 vs. 0.5 vs. 0.7 in transfer learning model

**Figure 5:**
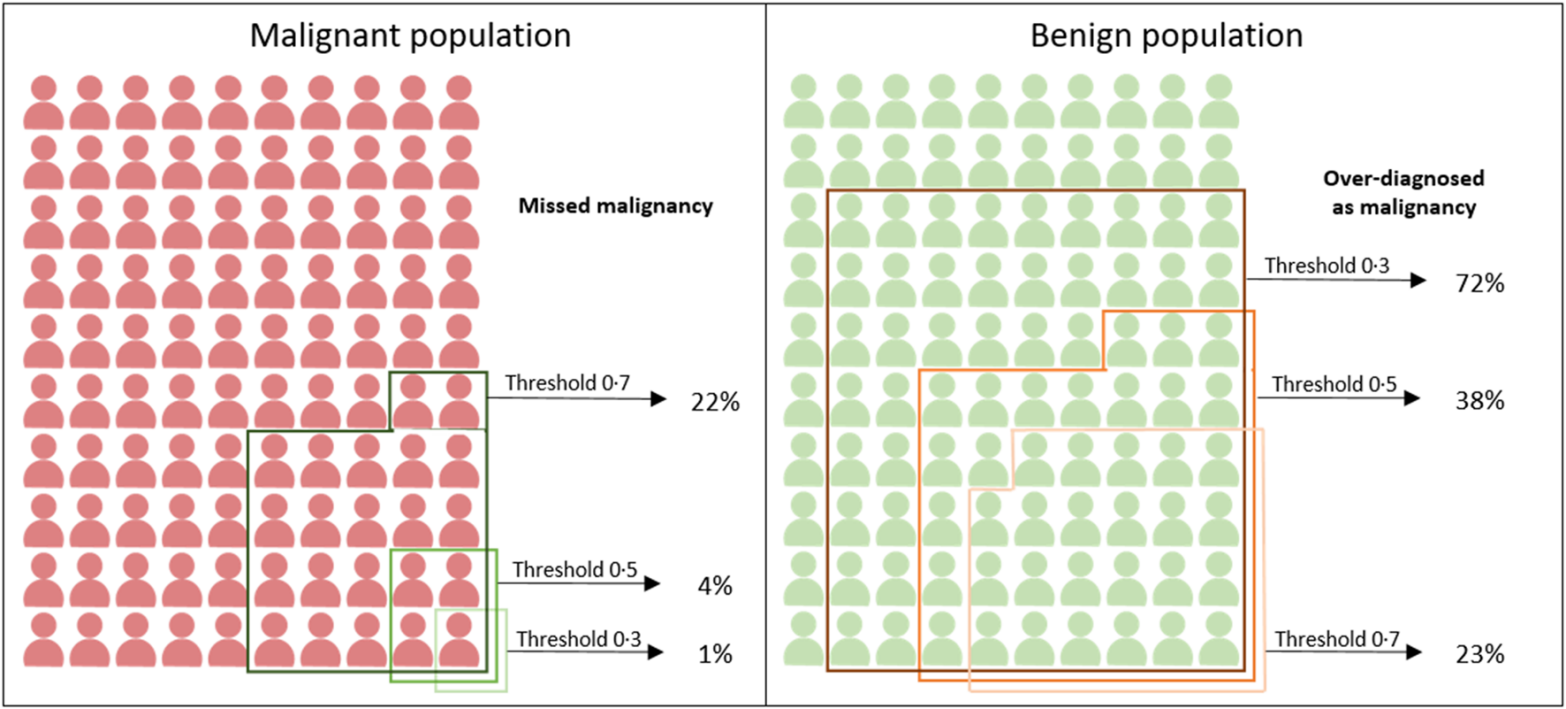
Performance of the algorithm according to the probability threshold changes. In malignant population (100 unit participant), there were missed malignancy prediction in 22%, 4% and 1% for threshold 0.7, 0.5 and 0.3, respectively. In benign population (100 unit participant), there were over-diagnosis as malignancy in 72%, 38% and 23% for threshold 0.7, 0.5 and 0.3, respectively.

Having confirmed that the transfer learning model outperformed the scratch model in most areas, we conducted a threshold test for the transfer learning model. The AUC values of the transfer learning model at probability thresholds of 0.3, 0.5, and 0.7 were the same for each dataset (Table 2). Inverse correlation to the probability threshold value was observed in sensitivity and NPV, whereas positive correlation was observed in accuracy (apart from the internal validation results), specificity, and PPV. Figure 5 illustrates the rate of false positive and false negative predictions according to the probability threshold changes in the malignant and benign populations. The numbers were postulated by summation of the five data set’s prediction results (test set of Set A and Sets B- E). The details for the respective data sets are shown in Supplementary Figs. 4–6.

**Table 2:** Summary of deep learning algorithm performance according to threshold value.

Dataset	Area under curve	Threshold	Negative predictive value
Internal test set A	0.889 ± 0.007	0.3	0.833 ± 0.031
		0.5	0.752 ± 0.035
		0.7	0.651 ± 0.036
External replication set B	0.775 ± 0.015	0.3	0.793 ± 0.038
		0.5	0.734 ± 0.035
		0.7	0.673 ± 0.026
External replication set C	0.781 ± 0.015	0.3	0.866 ± 0.03
		0.5	0.805 ± 0.048
		0.7	0.731 ± 0.043
External replication set D	0.809 ± 0.026	0.3	0.98 ± 0.018
		0.5	0.941 ± 0.032
		0.7	0.886 ± 0.031
External replication set E	0.905 ± 0.016	0.3	0.96 ± 0.041
		0.5	0.926 ± 0.056
		0.7	0.891 ± 0.055

In the malignant population (100 participant-unit), at thresholds 0.7, 0.5, and 0.3, malignancy was incorrectly predicted as benign in 22%, 4%, and 1% of cases, respectively. In the benign population (100 participant-unit), at thresholds 0.7, 0.5, and 0.3, benignity was incorrectly predicted as malignant in 72%, 38%, and 23% of cases, respectively.

## Discussion

According to our study results, the transfer learning model was more effective than the scratch model in training our deep learning algorithms to distinguish between benign and malignant thyroid nodules based on US thyroid images. Furthermore, we found that about 70% (3902 images) of the original dataset was the minimal proportion of images required for the deep learning algorithm to efficiently reproduce adequate training results. Finally, by altering the probability threshold value, it was possible to manipulate the algorithm performance to better suit the population characteristics while retaining overall performance.

There have been many studies on neural network models that stratify the risk of malignancy of thyroid nodule US images. Table 3 is a summary of several recent neural networks mostly trained with large datasets (exceeding 10,000 images) and a network trained with a small amount of data^18–22^, which illustrates how the accuracy of neural networks is affected by the volume of training data. In comparison, our deep learning algorithm included 4611 images and demonstrated an AUC of 0.889. These results are slightly lower than studies with more than 10,000 images, but superior to one study with 600 images. Therefore, our findings were in accordance with the published literature on this topic.

**Table 3:** Summary of recent studies on the thyroid ultrasound image analysis using neural networks.

Authors	Year	Architecture	Number of images	AUC	Accuracy	Sensitivity	Specificity
Wei et al.	2020	EDLC-TN	26,541	0.936–0.946	98.51	93.77	94.44
Peng et al.	2021	ThyNet	18,049	0.940–0.947	89.1	94.9	81.2
Koh et al.	2020	AlexNet-GoogLeNet-SqueezeNet-InceptionResNetv2	15,375	0.885–0.978	86	83.7	91.2
Bai et al.	2020	RS-Net	13,984	N/A	88	98.1	79.1
Zhu et al.	2021	VGG16	600	0.770–0.879	82	85	79

The current study has four significant aspects. First, this study demonstrates that the transfer learning model is more effective than scratch learning in training deep learning algorithms with US images of thyroid nodules. Transfer learning builds a model from the target domain (internal dataset) by exploiting information from the source domain (ImageNet) through a knowledge transfer processes^23^. Transfer learning is especially useful when training a new domain with data that are limited or too expensive to collect, which is generally the case with medical images. Using a transfer learning model via ImageNet, we were able to significantly improve the diagnostic performance of our deep learning algorithm compared to using the scratch model. Our findings are consistent with the existing literature on the efficacy of transfer learning for the differential diagnosis of benign and malignant thyroid nodules based on US images^12^.

Second, our stress test demonstrated that with 70% of the total dataset, it was possible to efficiently train the algorithm with US images of thyroid nodules. To train and tune our algorithm we used 5575 US images of thyroid nodules. Our stress test results suggest that 3902 images would be needed to economically reproduce a model with similar performance. The performance/accuracy of an AI model improves logarithmically (100-fold increase in performance accompanies a tenfold increase in data) based on the amount of data used in training^24^, and thus quantity matters more than quality^25^. In the field of medicine, however, it is difficult to indefinitely increase the number of images for training due to concerns regarding patient privacy and the labor intense expertise required in acquiring and preparing data^26^. In this regard, setting the minimum data required to feasibly predict the performance of the 100% dataset could be useful to future researchers. The 70% threshold should be interpreted with caution as the performance of a 100% dataset varies with the amount of data the model was initially trained with.

Furthermore, we demonstrated that it was possible to customize the optimal performance of the algorithm by adjusting the threshold probability without compromising overall performance. The primary purpose of thyroid nodule US is screening. When a confirmatory diagnosis of malignancy is required, or when US features are ambivalent, fine needle aspiration cytology results are required to derive a final diagnosis. In this regard, our deep learning algorithm should be tuned to enhance the screening ability of US. For a diagnostic screen, a higher false positive rate is better than a high false negative rate because it allows for follow-up fine needle biopsy to refute or confirm the diagnosis, allowing time for intervention. By lowering the probability threshold from 0.7 to 0.3, both the sensitivity and the NPV of the model improved by up to 20% while the AUC remained the same. Furthermore, to achieve its purpose as a screening tool, the sensitivity and specificity of the US must be adjusted depending on the estimated prevalence of malignancy in a population. Our findings indicate that by lowering the probability threshold to 0.3, the performance of an AI model may be tuned to better fit the purpose of US, which in this case is screening. Considering that the benign/malignancy ratios of Sets A to E do not reflect the true prevalence of thyroid cancer, such flexibility is crucial.

Finally, we attempted to overcome the issue of overfitting by acquiring external validation datasets for different institutions, including one from Kuma Hospital, Kobe, Japan. Overfitting describes a situation where a neural network learns statistical signals specific only to the training set and ends up learning insignificant noise rather than meaningful patterns, leading to decreased performance with new datasets^27^. Expanding the training set to include more data from various sources increases the model’s performance. Additionally, we have found that selection of architecture depends on not only the number of training dataset, but also complexity of input information^28^. We trained deeper version of algorithms such as ResNet 101, 152, InceptionV3, and a state-of-the-art algorithm such as ViT^29^. The AUC of ViTs with and without transfer learning was 85% and 75%, respectively, which were lower than that of VGG by 3% points and 4% points, respectively. As deep learning data analysis plays an adjunct role in the clinical field at least for now, such small difference may have little clinical significance. Also, this demonstrates that the latest version does not always show the best performance, and that the performance of an algorithm might not be dependent on the novelty of the algorithm, but on the quality or quantity of the input data. Furthermore, it is more important for the diagnostic tools demonstrate consistent performance than to show a bit higher but inconsistent performance in the clinical field. The most significant limitation of our study is that the total number of images were not as large as some of the recent publications reporting the development of neural networks that distinguish between benign and malignant US images of thyroid nodules^19^. However, we believe that our attempts to discover the minim data required, to avoid overfitting, and to customize the model to a screening setting compensate for the low volume of data.

## Conclusion

In conclusion, we proved that the transfer learning model was more effective in training our deep learning algorithm than the scratch learning model. Furthermore, we demonstrated that with 3902 images we were able to obtain an acceptable level of performance. With more data we will be able to train the algorithm to be more accurate.

## Supplementary Information

Supplementary Figures.

Supplementary Tables.

## Data Availability

The datasets generated during and/or analyzed during the current study are available from the corresponding author on reasonable request.
