# Supplementary material for: Investigation of optimal convolutional neural network conditions for thyroid ultrasound image analysis
Source: Sci Rep. 2023 Jan 24;13:1360. doi: 10.1038/s41598-023-28001-8 (PMC9873643; doi:10.1038/s41598-023-28001-8)
Supplement: Supplementary file 1 — Supplementary Figures. [file 41598_2023_28001_MOESM1_ESM.docx]

*
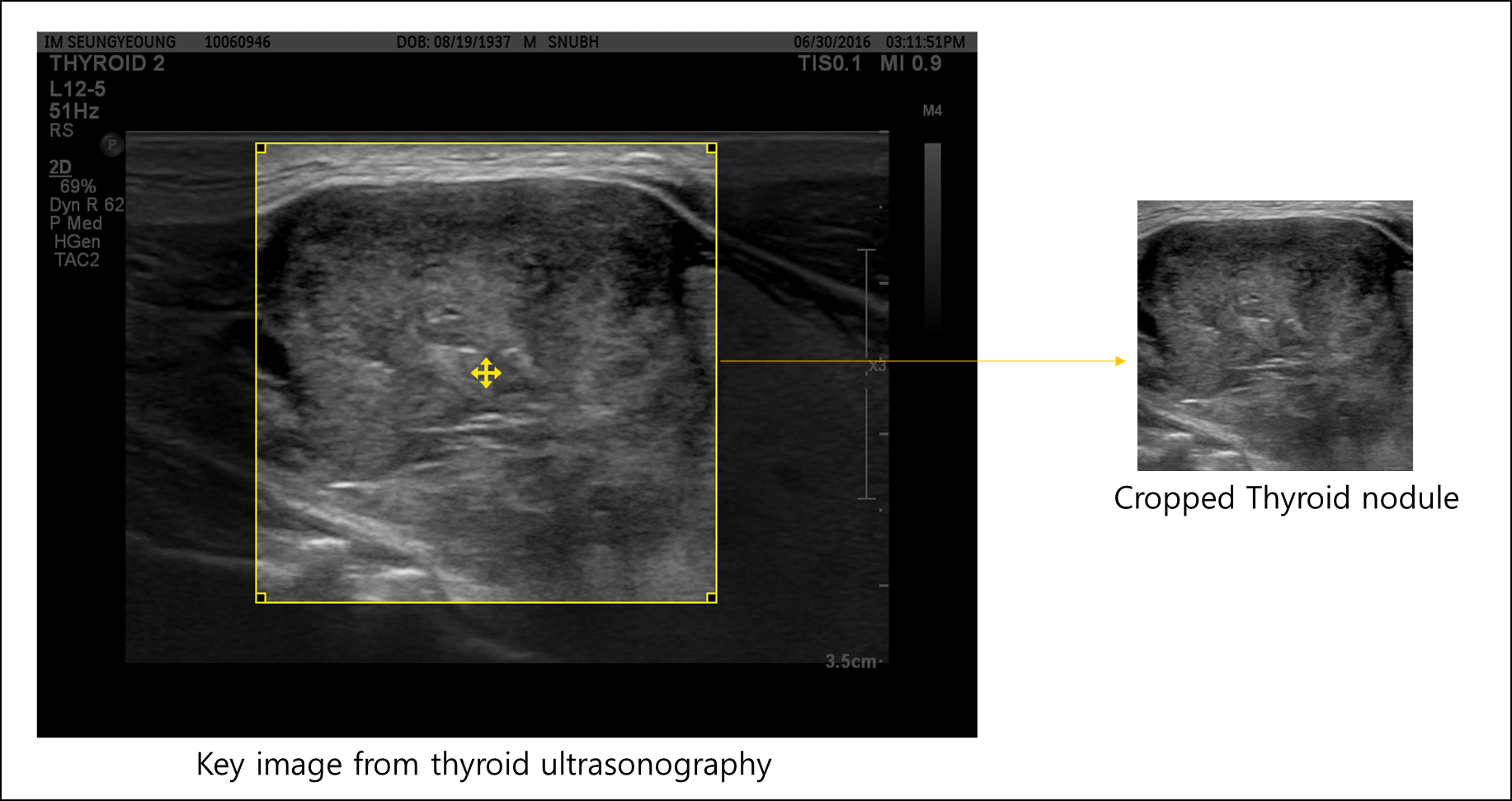
*

**Supplementary Figure 1. Cropping the region of interest from the thyroid US image** The yellow box is adjusted to place the thyroid nodule in center. Once the yellow box is placed, “save cropped image” function will save the cropped image automatically.


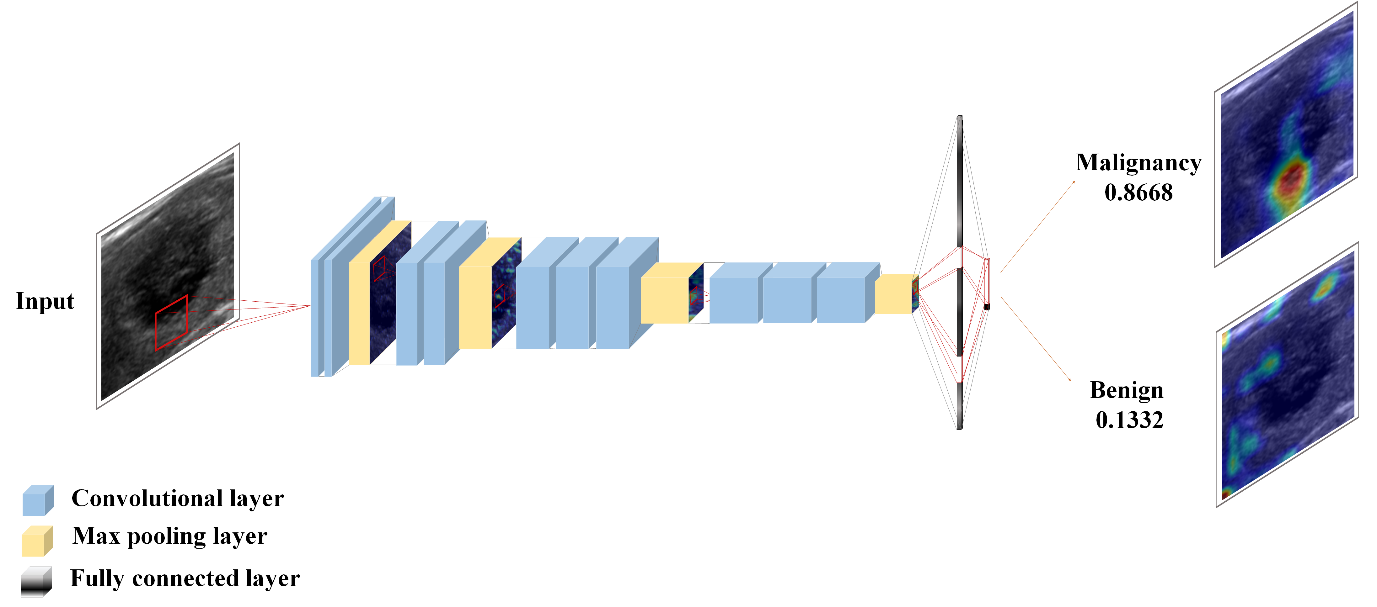
**Supplementary Figure 2. General schematic map of convolutional neural network architecture**


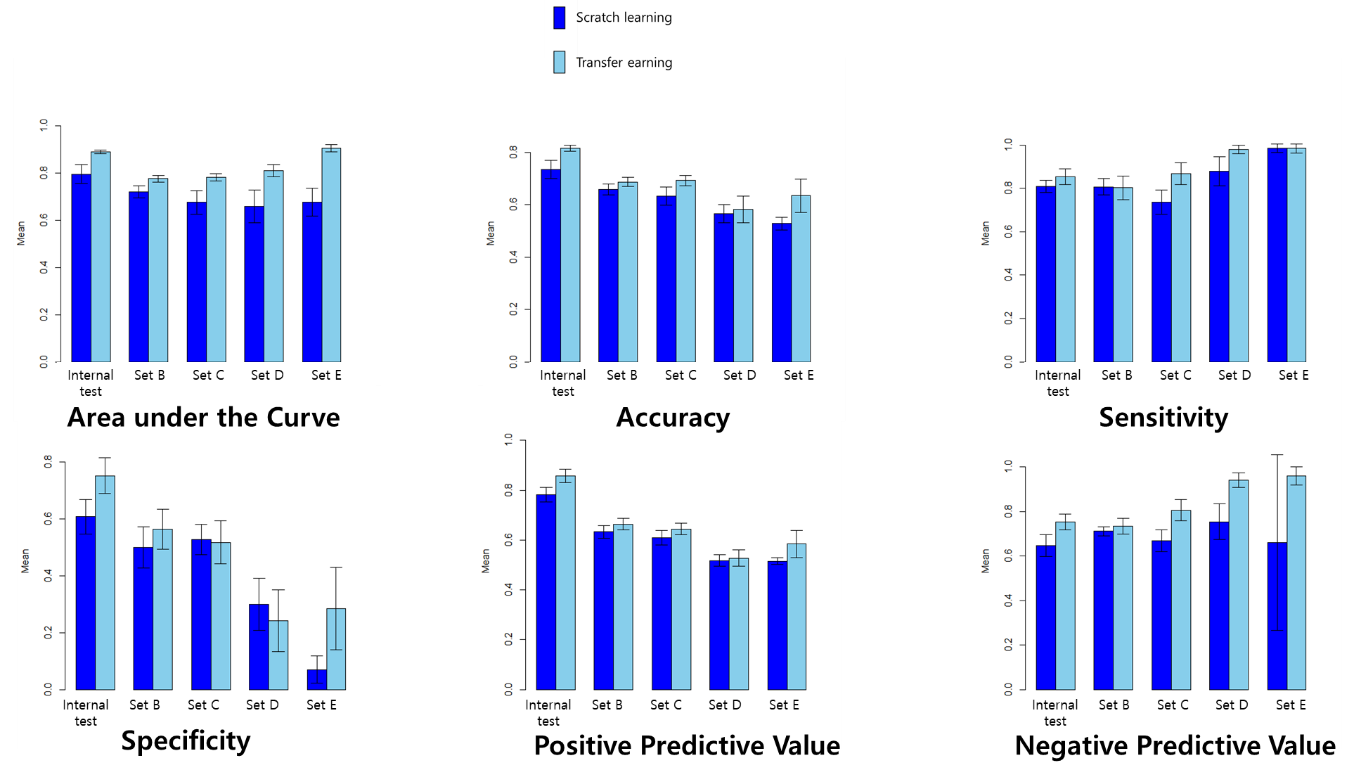


**Supplementary Figure 3. Comparing the performances between scratch learning versus transfer learning in 5 data**

**
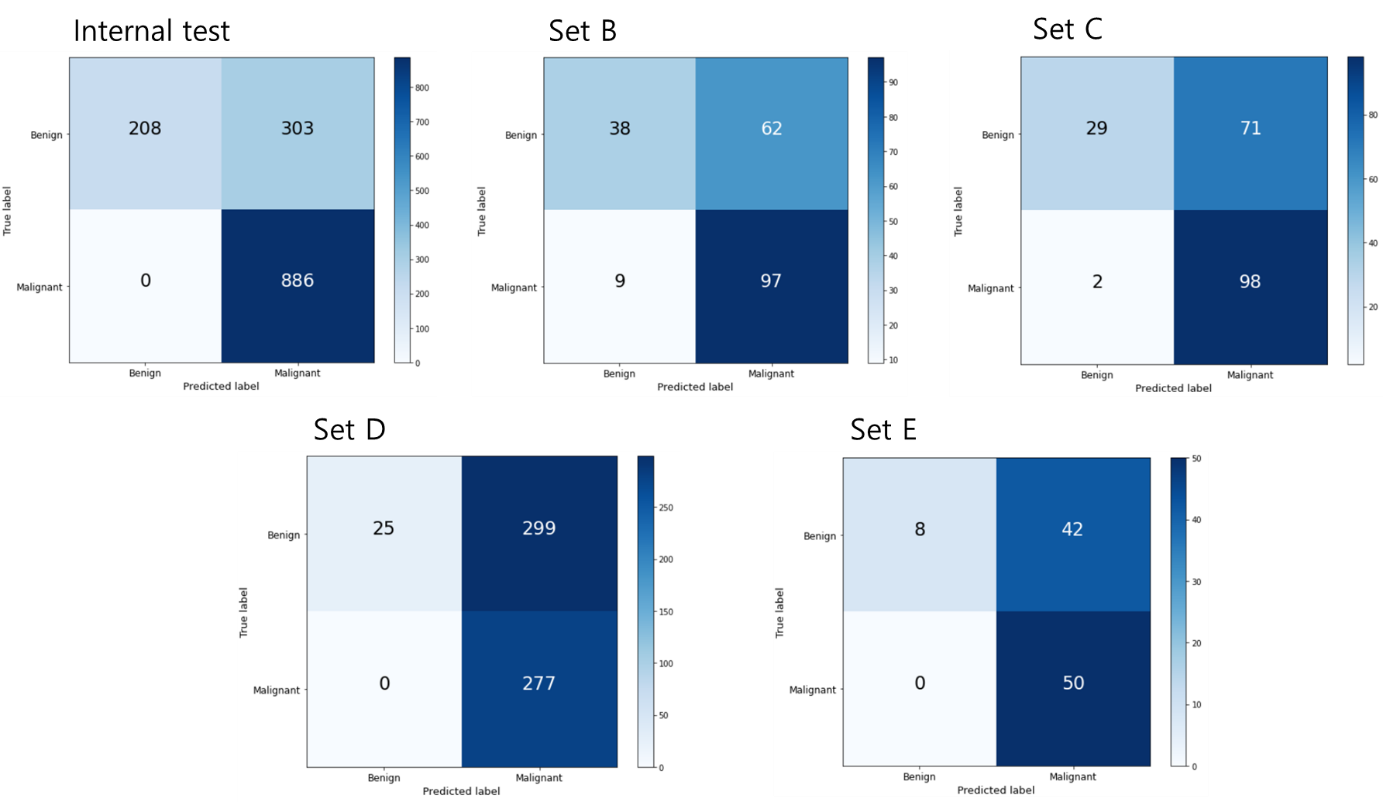
**

**Supplementary Figure 4. Illustration of the missed malignancy and over-diagnosis as malignancy rate according by probability threshold 0.3 using VGG19**

**
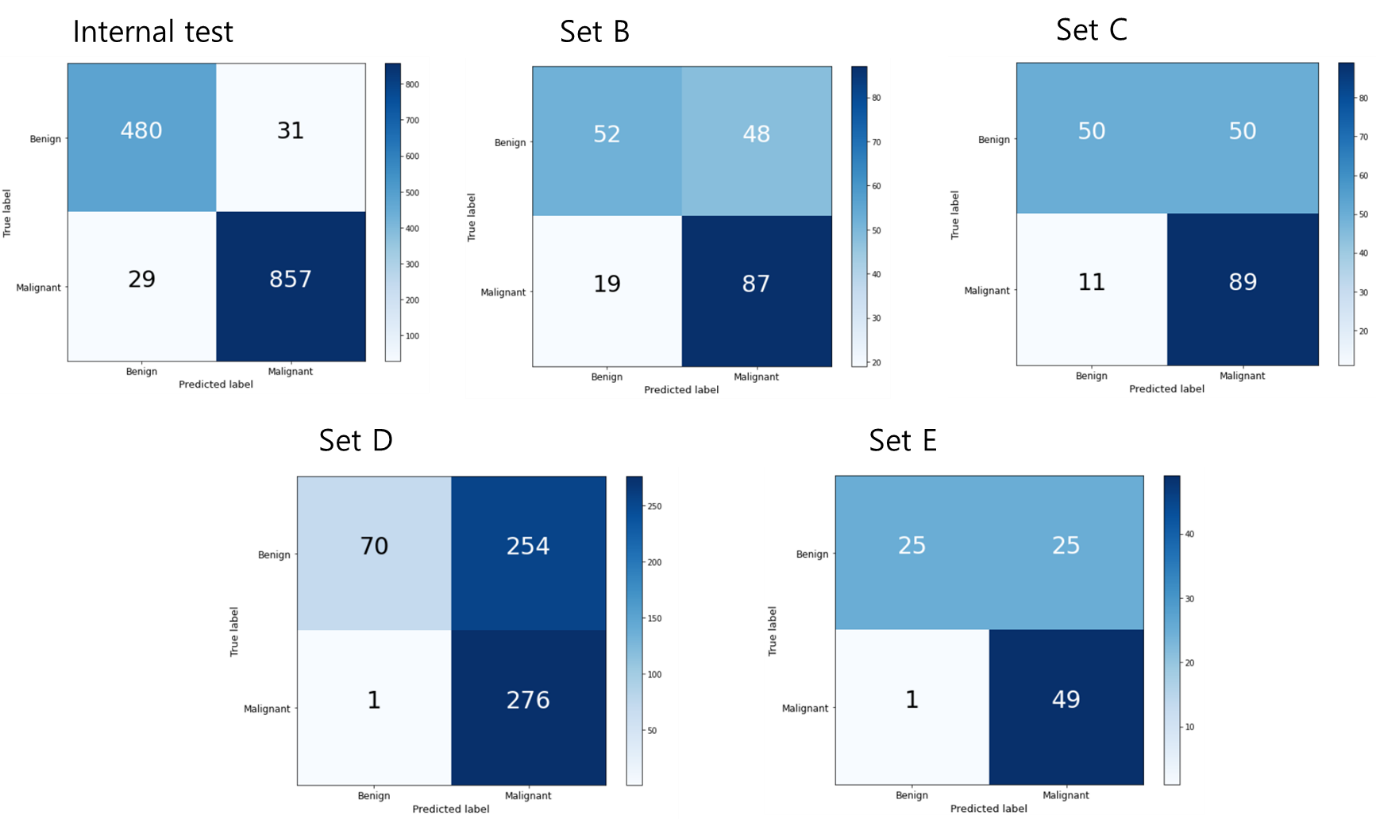
**

**Supplementary Figure 5. Illustration of the missed malignancy and over-diagnosis as malignancy rate according by probability threshold 0.5 using VGG19**

**
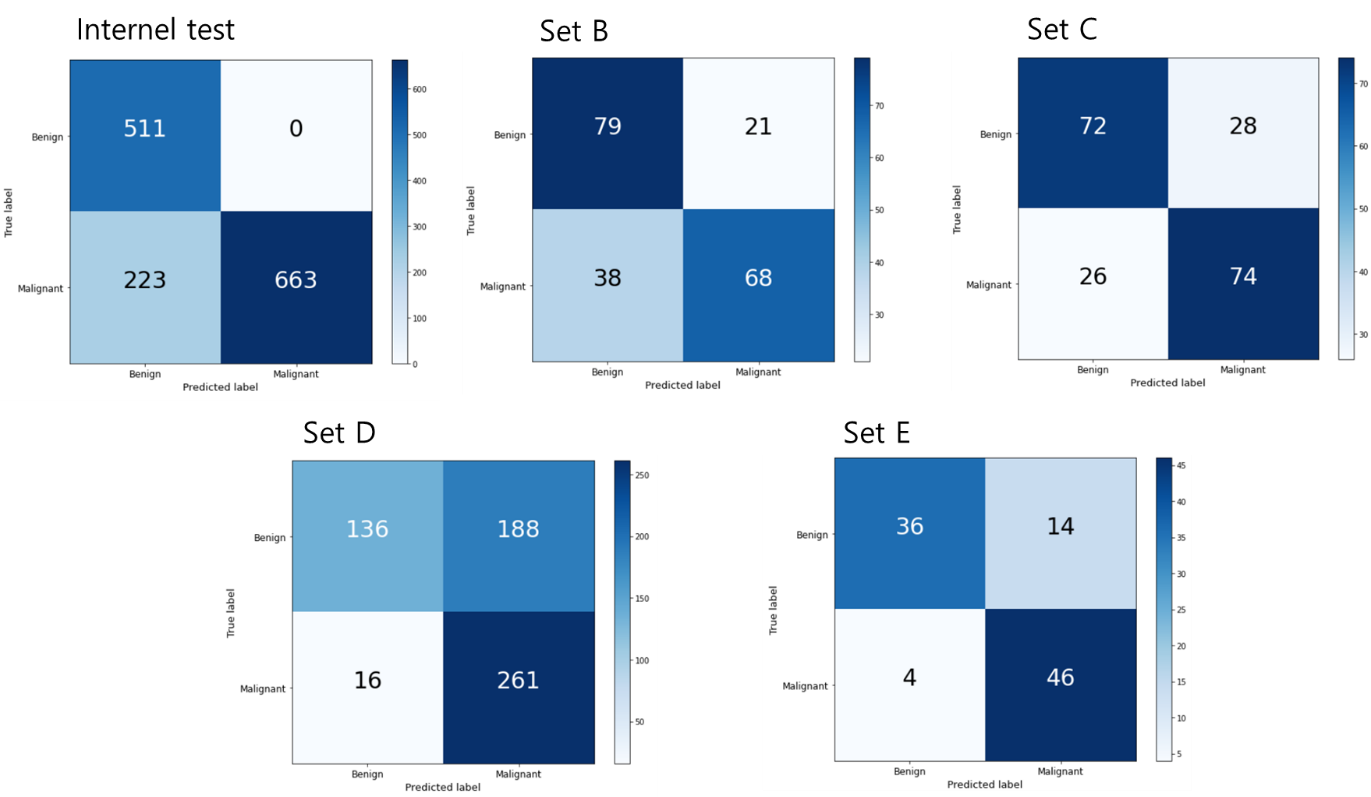
**

**Supplementary Figure 6. Illustration of the missed malignancy and over-diagnosis as malignancy rate according by probability threshold 0.7 using VGG19**
